# Condition Monitoring of a Three-Cathode Cascaded Plasma Spray Torch Regarding Process Reliability

**DOI:** 10.3390/ma15186203

**Published:** 2022-09-06

**Authors:** Georg Mauer, Frank Kurze, Karl-Heinz Rauwald, Robert Vaßen

**Affiliations:** Forschungszentrum Jülich GmbH, Institute of Energy and Climate Research, IEK-1: Materials Synthesis and Processing, 52425 Jülich, Germany

**Keywords:** plasma spraying, cascaded plasma torch, three-cathode plasma torch, process reliability, substrate surface temperature

## Abstract

The TriplexPro™-210 plasma spray torch (Oerlikon Metco) is a three-cathode plasma generator. It became a kind of workhorse for the wide range of tasks handled at the Jülich Thermal Spray Center (JTSC). Compared to conventional single-cathode torches, the cascaded design of the nozzle suggests low fluctuations of the arc and thus high stability. However, after a certain time, degradation sets in even with such a torch, impairing the reliability of the process. It is therefore important to detect indications of performance loss in time and not only during the inspection of the deposited layer. In this study, standard samples of YSZ thermal barrier coatings were sprayed regularly over a period of two years. Operational data and feedstock characteristics were collected and correlated with the area-specific mass deposition. It turned out that the measured substrate surface temperature showed a distinct correlation. Searching for the reasons for the temperature variations, several process parameters could be ruled out as they are monitored by calibrated sensors, controlled, and their time course is recorded by the control unit. Moreover, there are other parameters, which can have a considerable impact such as the robot alignment or the substrate cooling conditions. However, the purposeful experimental variation of such variables resulted in a variability of the mass deposition being considerably smaller than observed over the two years. Thus, it can be concluded that torch degradation had a pronounced effect, too. The substrate surface temperature can be used as indicator for the torch status and the reliability of the spray process.

## 1. Introduction

*Reliability* means that a system operates as expected over a specified timeframe. For a thermal spray system, reliability corresponds to the probability of manufacturing a product successfully and consistently with respect to specific requisite service properties. As such, this term covers all aspects of *repeatability* and *reproducibility* [1].

Monitoring and improving of process reliability are prevalent issues in thermal spray technology [2]. This is extremely challenging since these processes involve a large number of variables, some of them not being well controlled such as wear of the electrodes in plasma generators [3]. Hence, it is a widely accepted industrial praxis to spray standardized test samples and to evaluate their coating characteristics by non-destructive testing methods such as thickness and hardness measurements, or destructive tests such as inspection of metallographic cross-sections, bending, or adhesion tests.

One way to spray test samples are static spray spots or cones. They were described already three decades ago [4,5]. Static spray spot analyses can be used, e.g., to adjust the powder injection and to align multiple injectors relative to the hot plasma core with the objective of optimum particle heating and an orientation-independent footprint of the plume [6]. Additionally, coating formation was investigated by observation of particle impacts during spraying of such cones [7]. Another approach of a spray test is the in-situ curvature analysis [8,9]. Here, a thin cantilever is coated, and the development of its curvature is measured during deposition and cooling.

Spray tests are done periodically or at particular opportunities such as changes of spray jobs and feedstock batches or after maintenance and replacement of equipment components [6]. Spraying such test samples is an appropriate approach for process monitoring and detection of deviations since all relevant process variables are covered and coating characteristics are determined directly [2]. At Jülich Thermal Spray Center (JTSC) [10], plane specimens are sprayed with a standard material at specified conditions in regular intervals to monitor the process reliability. Thereby, the area specific coating weight proved to react sensitively to process deviations in coating mass.

This study was triggered by the variability of coating mass deposition over time obtained on the test samples sprayed with a three-cathode cascaded plasma torch in the time period 2020 and 2021. To find reasons, the variabilities of the particle size distributions, the plasma torch net power, the torch voltage, and the sample surface temperature were evaluated during the coating process. Since the latter turned out to be a suitable indicator of variations in the deposition rate, further spray experiments were conducted with targeted variation of parameters which are typically not monitored by calibrated sensors, automatically controlled, and logged [2] to investigate their effects. The importance of monitoring the coating temperature during the spray process has been emphasized already by several authors [11,12]. The methods applied [13,14] were infrared (IR) pyrometry [15], IR thermography [16], and thermocouples [17].

## 2. Materials and Methods

Test samples were manufactured from mild steel substrates (AISI 1015) with dimensions of 50 mm × 50 mm × 1.5 mm. These were grit-blasted with an air pressure of 0.25 MPa using high-grade corundum with a F36 grain size (425–600 µm), which led to an arithmetic mean roughness of *R_a_* = 3.7 ± 0.3 µm. The deposited coatings consisted of ZrO_2_ partially stabilized by 8-wt% Y_2_O_3_. The powder type was 204NS (Oerlikon Metco, Westbury, NY, USA) with a median diameter *d*_50_ = 55.7 ± 2.6 μm (measured by laser diffraction, LA-950, Horiba Europe, Oberursel, Germany). It was fed at a rate of 31 g min^−1^; the carrier gas flow was 2 slpm argon. Coating porosities were determined on SEM images of polished cross-sections by digital image analysis using the ImageJ software [18].

The atmospheric plasma spray process was performed using a three-cathode DC plasma torch with a cascaded nozzle (TriplexPro™-210, Oerlikon Metco, Wohlen, Switzerland) mounted on a six-axis industrial robot. In this gun, three single arcs are generated which are forced to have a fixed length by inserting a stack of neutrodes in front of the anode. The arcs behave more stable than in legacy non-cascaded torches, as the fluctuations of the anode attachments in axial and azimuthal directions are limited [19]. Thus, the power demand is virtually constant, and the feedstock particle treatment is uniform. Furthermore, the lifetime of the electrodes is enhanced as the power is split to three arcs so that the power density is decreased. In this work, a standard parameter set for porous thermal barrier coatings with a plasma gas mixture of 46 slpm argon and 4 slpm helium and a current of 420 A was used. This resulted in an electrical input power of 37.4 kW.

Using the TriplexPro™-210 torch, the azimuthal position of the powder injectors is step-adjustable by 20° between 0° and 80° to improve particle injection into the non-rotationally symmetric plasma jet. In this work, the injectors were mounted at the 40° position, so that the injection took place between the three hot cores of the plasma jet and the powder particles were effectively held in the jet (so-called cage effect [20]). This position was found in a previous work [21]. The spray distance was 200 mm, and the robot travel velocity was set at 500 mm s^−1^, while the meander dimensions were 150 mm × 2 mm × 25 mm. The coatings were deposited in 14 cycles to obtain a total coating thickness of approximately 400 µm.

Three preheating passes were performed before spraying. The samples were cooled on the front side by compressed air at 0.4 MPa. The sample surface temperature during the coating process was measured by a single-wavelength pyrometer (M8, Land Instruments Int., wavelength 8–14 µm, observation spot diameter 10 mm, 0–95% response time 100 ms). In this wavelength range, there are no emission lines of neutral or single-ionized argon; for Ar I, the electron transition with the lowest energy difference corresponds to an emission wavelength of 2.4 µm; for Ar II, the largest emission wavelength is 7.0 µm [22]. Generally, pyrometers operating at wavelengths >6 µm are considered insensitive to radiation of the plasma and the hot particles under thermal spray conditions [23].

## 3. Results and Discussion

**Figure 1:**
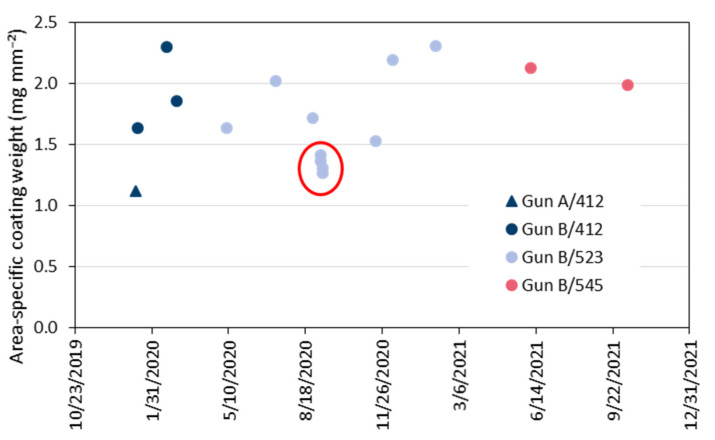
Variability of the area-specific coating weights on the sprayed test samples obtained in the period of 2020 and 2021 (see text for details).

This work was triggered by the variability over time of the area-specific coating weights obtained on the sprayed test samples. Figure 1 shows the values for the years 2020 and 2021. In the first test, a very low coating weight of 1.12 mg mm^−2^ was obtained. Thus, the gun (hereafter referred to as gun ‘A’) was replaced by a completely reworked torch with new electrodes (hereafter referred to as gun ‘B’). The next two tests with a time interval of 38 days gave coating weights of 1.63 mg mm^−2^ and 2.31 mg mm^−2^, respectively. It is well known that a training effect can occur if a new gun is commissioned. After four more tests with the powder batch #412, a new powder batch #523 was started in May 2020. After three tests on almost normal level, a very poor coating weight was observed at 1.42 mg mm^−2^. This test was repeated, then the powder hopper was revised, and finally a new powder bottle was started. However, the coating weights obtained in these three additional tests remained also on low level between 1.27 mg mm^−2^ and 1.31 mg mm^−2^. During the next three tests until February 2021, coating weights increased again to normal level of approx. 2 mg mm^−2^. Another change of the powder batch from #523 to #545 virtually did not affect the coating weights.

### 3.1. Variability of Dedicated Process Parameters

#### 3.1.1. Variability of the Powder Particle Size Distribution

**Figure 2:**
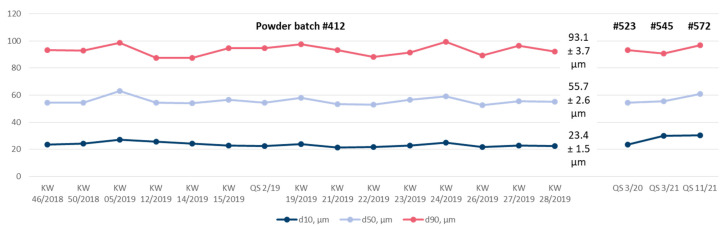
Variability of the particle size distribution obtained in the period of 2020 and 2021 (see text for details).

During the starting phase of the tests in 2020, the particle size distribution of the used powders was measured almost every week by laser diffraction. Figure 2 shows the results. As long as the first powder batch #412 was in use, d_10_ was 23.4 ± 1.5 µm, d_50_ was 55.7 ± 2.6 µm, and d_90_ was 93.1 ± 3.7 µm (mean and standard deviation). No abnormalities are apparent in the curves. Thus, it was decided to extend the time intervals of the particle size measurements to a quarterly basis. The change of powder batches from #412 to #523, #545, and #572 in the subsequent periods did not result in distinct deviations from the initial characteristics. Hence, the supposed variability of the powder particle size could be ruled out as a reason for the observed variation of the coating weights.

#### 3.1.2. Variability of the Net Power

The plasma torch net power is defined as the electrical input power minus the power dissipated by the water-cooling. The input power is simply obtained by multiplying the torch current and the voltage. Both are measured continuously and tracked by the control unit at a sampling rate of 1 Hz. Then, the net power can be calculated based on the cooling water flow, the specific heat of water, and the temperature difference between feed and return. These values are also recorded by the controller so that the cooling power can be determined hereafter if it was not read from the display and logged during coating.

**Figure 3:**
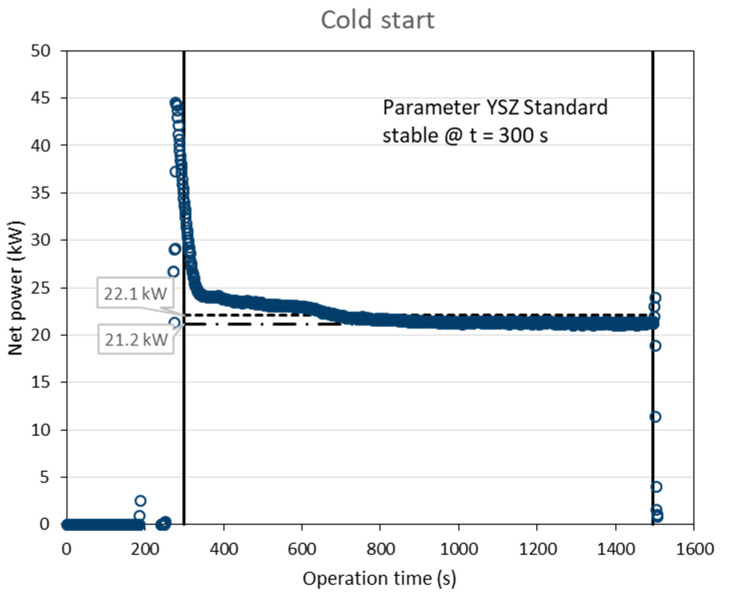
Time course of the torch net power calculated from the torch input power and the power dissipated to the water cooling (cold start of the gun); stable plasma conditions were indicated by the controller at *t* = 300 s.

**Figure 4:**
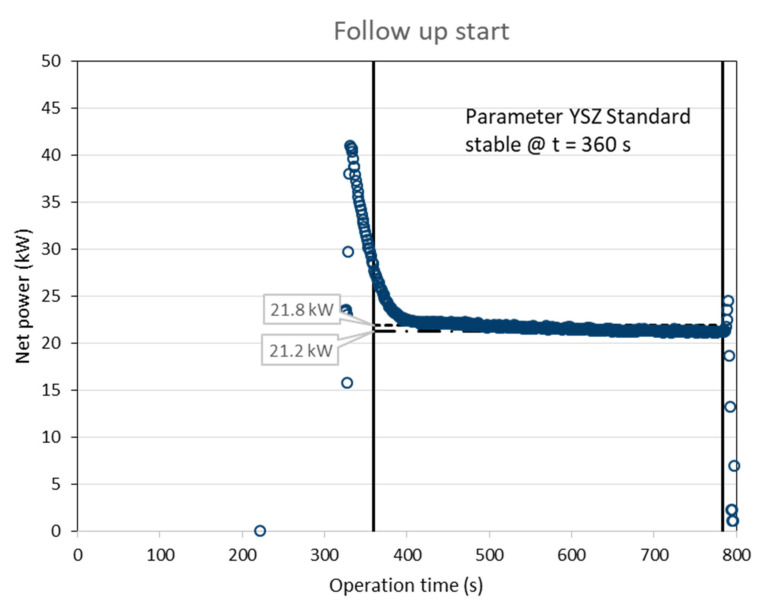
Time course of the torch net power calculated from the torch input power and the power dissipated to the water cooling (follow up start of the gun); stable plasma conditions were indicated by the controller at *t* = 360 s.

Figure 3 gives the time course of the torch net power calculated from the torch input power and the power dissipated to the water-cooling after a cold start of the gun. Stable plasma conditions were indicated by the controller at *t* = 300 s. It is obvious that the cooling system needed longer time to reach stable temperatures and thus a constant cooling performance. The final net power before shutdown of the gun was 21.2 kW, while the average over the coating time was 22.1 kW. Figure 4 shows the time course after a follow up start of the gun. Here, stable plasma conditions were indicated by the controller at *t* = 360 s. The final net power before shutdown of the gun was again 21.2 kW, while the average over the coating time was 21.8 kW. Obviously, the most reliable way to determine the torch net power is to log the final value just before shutting down of the gun.

**Figure 5:**
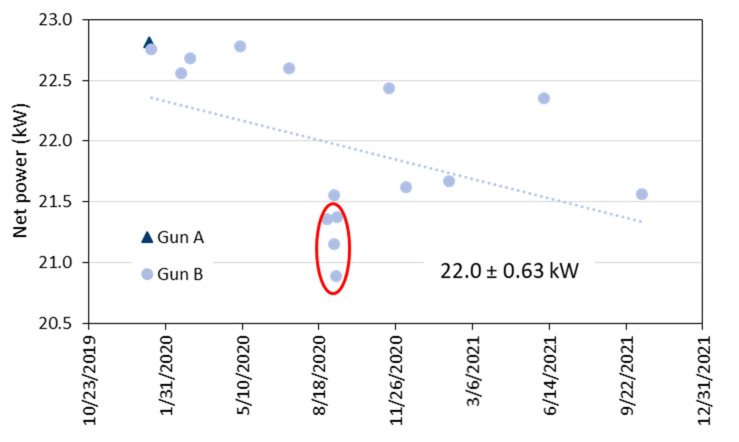
Variability of the torch net power obtained in the period of 2020 and 2021.

**Figure 6:**
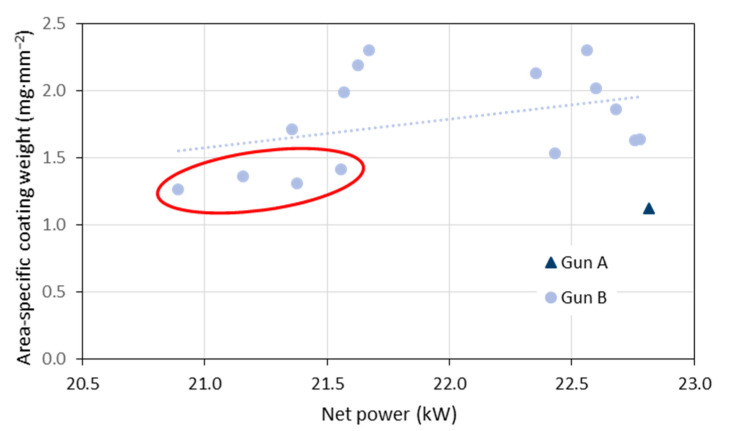
Area-specific coating weights plotted against the torch net power.

In Figure 5, the variability of the torch net power obtained in the period of 2020 and 2021 is given. The mean was 22.0 kW and the standard deviation 0.63 kW. Figure 6 shows the area-specific coating weights plotted against the torch net power. Similar results were obtained if the torch efficiency (net power divided by input power) was considered instead of the net power. It is obvious that the four samples with the lowest coating weights (marked with the red ellipses) were sprayed at the lowest torch net power. Hence, the net power could be a reliable indicator for problems with the deposition efficiency. This was also stated by Leblanc et al. [24]. In contrast to that, the very first test sample sprayed with gun ‘A’ also showed a poor coating weight was sprayed at the highest net power of 22.8 kW. Thus, the net power cannot be a unique indicator as far as different hardware is considered. A considerable hardware dependence of the in-flight particle temperature was reported already by Moreau [25] who used three nominally identical plasma spray guns.

#### 3.1.3. Variability of the Voltage

**Figure 7:**
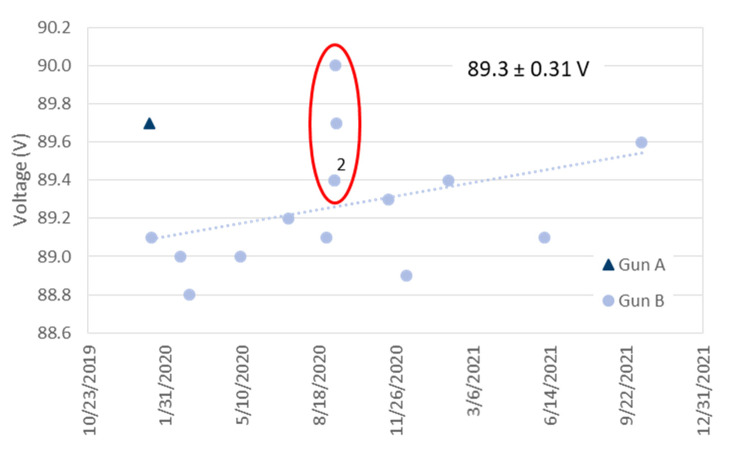
Variability of the torch voltages measured in the period of 2020 and 2021 (‘2’ indicates that one data point is hiding another one).

**Figure 8:**
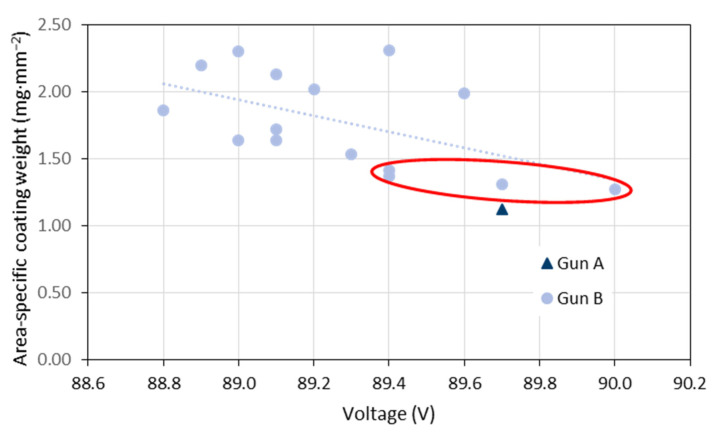
Area-specific coating weights plotted against the torch voltage.

Spray systems are usually operated in constant current mode. This means that the voltage is adjusted by the controller depending on the plasma gas flow and composition as well as on the impressed current. As mentioned above, the voltage is also recorded by the controller at a sampling rate of 1 Hz and averaged over the coating time. Since a three-cathode torch was used, it is also an average value from the corresponding three individual voltages. Figure 7 gives the variability of these torch voltages observed in the period of 2020 and 2021. The mean was 89.3 V and the standard deviation 0.31 V. In Figure 8, area-specific coating weights are plotted against the torch voltage. Similar results were obtained if the torch input power was considered instead of the voltage. The very first sample sprayed with gun ‘A’ and two of the four samples with the lowest coating weights (gun ‘B’, marked with the red ellipses) were sprayed at the highest voltages at 89.7 and 90.0 V. However, for the other two samples the voltage increase is not that clear. Obviously, the torch voltage is only partly suitable as indicator of problems with the reliability.

#### 3.1.4. Variability of the Sample Surface Temperature

**Figure 9:**
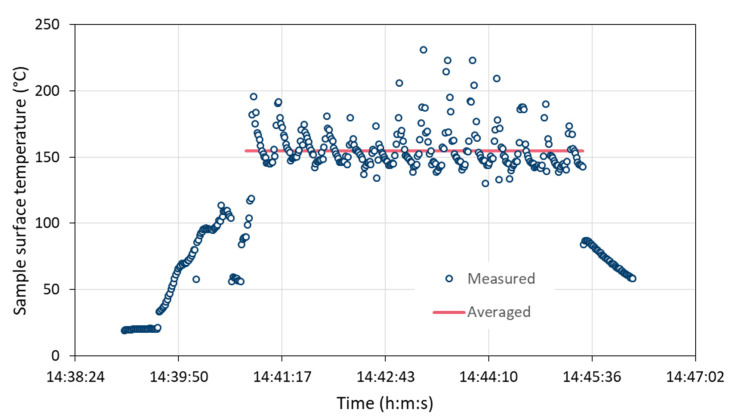
Example for measured and averaged sample surface temperature during coating deposition.

It is well known that one of the main parameters affecting the characteristic attributes of thermally sprayed coatings is the temperature of the workpiece [26]. During the tests, the sample surface temperature during coating deposition was continuously measured by a pyrometer and recorded by the control unit of the spray system at a sampling rate of 1 Hz. The line of sight between pyrometer and sample is interrupted sometimes by the moving gun. Due to the interference of the torch movement and the sampling frequency, it is uncertain whether the maximum temperature during each coating cycle is always captured. Thus, the temperatures averaged over all cycles were evaluated. Figure 9 gives an example of the time course. The three heating and 14 coating cycles are clearly visible. Only the data points during coating deposition were considered for averaging.

**Figure 10:**
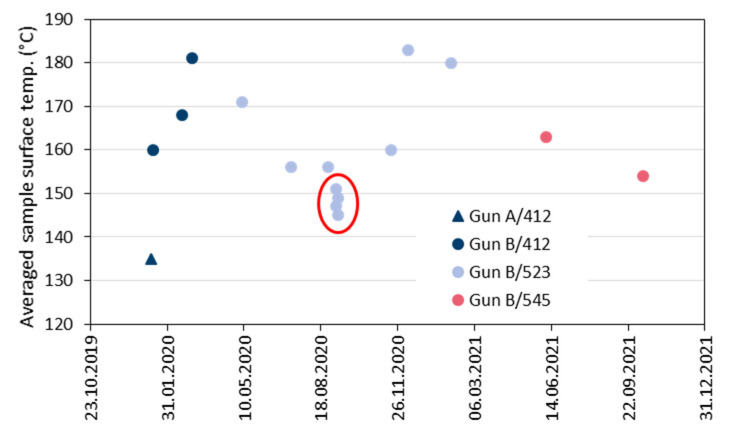
Variability of the averaged sample surface temperature evaluated in the period of 2020 and 2021; the legend indicates the gun in use and the designation of the powder batch.

**Figure 11:**
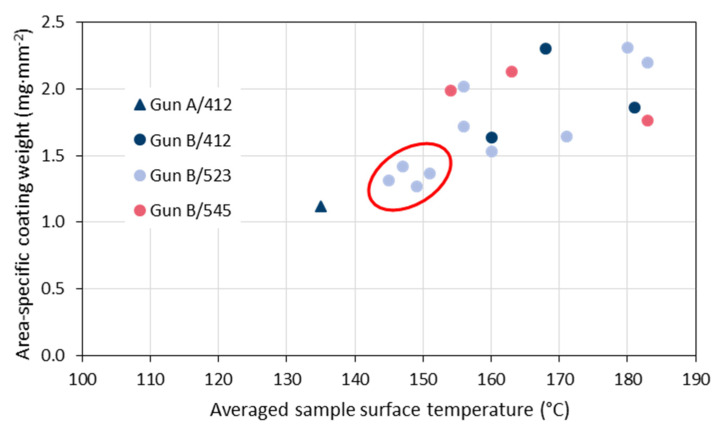
Area-specific coating weights plotted against the averaged sample surface temperature observed in 2020 and 2021; the legend indicates the gun in use and the designation of the powder batch.

Figure 10 gives the variability of the averaged sample surface temperature evaluated in the period of 2020 and 2021. It is evident that the very first test sample sprayed with gun ‘A’ and the four data points with the poorest coating weights sprayed with gun ‘B’ (marked by the red ellipse) revealed the lowest surface temperatures. In Figure 11, the area-specific coating weights are plotted against the averaged sample surface temperature. Obviously, there is a quasi-linear correlation between these two effects of the coating process. As the data points for the three used powder batches #412, #523, and #545 are well distributed, it is confirmed that they did not have any significant effects. These results suggest that the averaged measured sample surface temperature is a suitable indicator for problems with the process reliability even if different spray hardware is used.

### 3.2. Possible Effects on the Sample Surface Temperature

These results raise the question of what might be the reasons for the variation of the sample surface temperature between 135 °C and 183 °C in 2020 and 2021. At the same time, the variability of the area specific coating weight was 1.12–2.31 mg mm^−2^. Some process parameters could be ruled out as they were monitored by calibrated sensors, controlled, and logged by the control unit of the spray system (plasma gas flow and composition, current, powder feed rate, carrier gas, and cooling air flow) or the robot controller (meander dimensions, constant spray distance, gun travel velocity, and number of coating cycles). However, there are some additional parameters which could affect the process variability. These are not covered by the data recording and monitoring of the controller and do not have any impact on measured particle in-flight characteristics (velocities, temperatures) which are often suggested for monitoring process reliability. To investigate their effects, they were deliberately varied in a series of 14 spray experiments:

- Standard setup start
- Without cooling at all/with max. cooling 5 bar front + back
- Smaller substrate 48 × 48 mm^2^/larger substrate 52 × 52 mm^2^
- Cooling nozzles aligned to crossing point at spray distance +20 mm/parallel
- Spray distance to sample −2 mm/+2 mm
- Meander shifted 10 mm left/right
- Meander shifted 5 mm up/down
- Standard setup end

**Figure 12:**
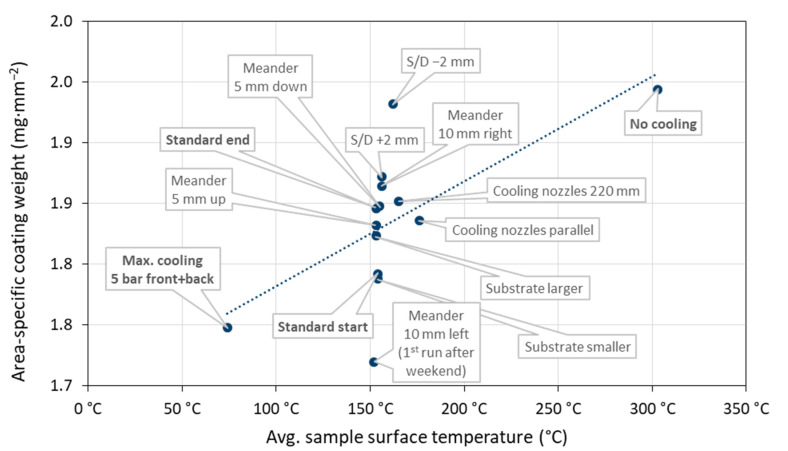
Area-specific coating weights plotted against the time-averaged surface temperature for a series of 14 spray experiments with deliberately varied process parameters.

Gun ‘B’ was used having an operation time of 80.7 h and 619 ignitions at the end of these tests. Figure 12 gives the corresponding area-specific coating weights plotted against the time-averaged surface temperature. On the one hand, the sample surface temperature varied in this set of experiments between 74 and 308 °C, which is much more than in 2020 and 2021. Here, the greatest influence was exerted by the cooling conditions (intensity, cooling nozzle alignment). On the other hand, the variability of coating weights was 1.72–1.94 mg∙mm^−2^ which is much smaller than observed in 2020 and 2021. Thus, it is obvious that the varying sample surface temperature cannot be the only reason for the reliability problems in the coating deposition in 2020 and 2021. There must have been at least one other significant reason.

This can be degradation of the torch, namely of the electrodes. Wear of the torch electrodes is an important reason for process deviations. It can be detected only by monitoring trends with respect to a known reference state. Since the formation of wear is dependent on the torch load spectrum, general thresholds for specific parameters are difficult to define [2]. It is hard to assess by visual inspection whether the electrodes are worn actually, or just show traces of use. By wear, the electrode surface condition can change so that the attachment, root movement, and resistance of the arc are affected [27,28]. Films can form that can be even removed later again during further torch operation. This may be an explanation for the recovery of the torch performance observed at the end of 2020. Thus, torch degradation by unnoticed wear of the electrodes was assumed the reason for the temporary drop of the coating weights in 2020 and 2021.

This makes it even more important to have a robust criterion for assessing the torch status in the form of the sample surface temperature during coating. It revealed a quasi-linear correlation with the deposited coating weights as shown above in Figure 11. In fact, it was suggested more than 20 years ago to include the pyrometrical measurement of the substrate temperature in control strategies to detect drift in powder injection conditions and torch working parameters of plasma spray processes [29,30].

**Figure 13:**
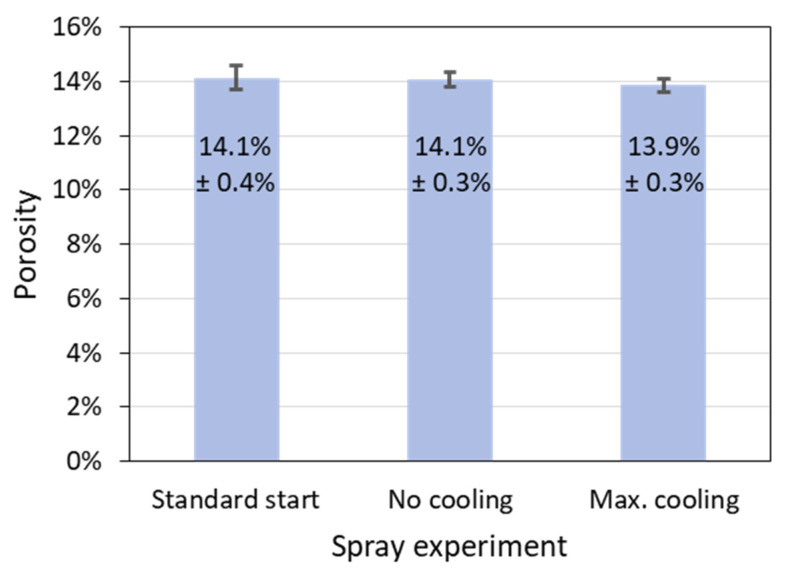
Porosities determined by image analysis of the two samples without and with maximum cooling as well as of the standard sample; the error bars denote the standard deviations of the porosities obtained from five images in each case.

In this context, it is interesting to note that porosity as another possible indicator of the process reliability turned out not to be meaningful in this work. In Figure 13, the porosities determined by image analysis of the above-mentioned two samples without any and with maximum cooling are shown as well as the porosity of the standard sample for comparison. The differences of the porosities are very small and not significant, although varying the cooling had resulted in the largest difference in the area-specific coating weights (see Figure 12).

## 4. Conclusions

During regularly spraying standard samples over a period of two years, temporary drops in the deposited coating mass were observed. Operational data and feedstock characteristics were collected and evaluated. It turned out that the measured substrate surface temperature showed a distinct correlation even if two different spray guns were used. Searching for the reasons, torch degradation came into focus. Accordingly, the substrate surface temperature is suggested as a reliable criterion to assess the torch status and to detect reliability problems with respect to the deposition efficiency and coating characteristics at an early stage.

It is assumed that the degradation of the torch namely of the electrodes has an impact on the plasma enthalpy and the heating of the feedstock particles [28]. Hence, the energy transferred to the substrate can be affected [24]. Both the plasma gas stream and the hot particle flow contribute to this energy transfer [17]. Thus, it is reasonable that torch degradation could result in variations of the sample surface temperature during coating.

The results of this study confirm the demand for more data that must be covered by modern process monitoring systems and capable methods needed to interpret them appropriately [3].

## Data Availability

The data presented in this study are available in the article.
